# Technology Acceptance from Above and Below. Negotiations of Energy Saving, Statehood, and Distributive Fairness in West Germany, 1976–1986

**DOI:** 10.1007/s00048-026-00446-8

**Published:** 2026-03-12

**Authors:** Thomas Lettang

**Affiliations:** 1 Department of History, Humboldt-Universität zu Berlin, 10099 Berlin, Germany; 2 Leibniz Centre for Contemporary History Potsdam (ZZF), Am Neuen Markt 1, 14467 Potsdam, Germany

**Keywords:** Energy saving, Policy, Social compatibility, Technology assessment, Participation, West Germany, Energiesparen, Regulierung, Sozialverträglichkeit, Technikfolgenabschätzung, Bürgerbeteiligung, Westdeutschland

## Abstract

In the mid-1970s, politicians and regulatory experts considered that the West German energy system was at a crossroads. They discussed whether energy saving and renewable energies or nuclear energy should be supported as a long-term technology. The debate on technology acceptance was not only sparked by questions of economic efficiency and the risks of nuclear energy use. It was also shaped by the dispute over regulatory instruments, consumer participation, and the organization of the energy system. The example of energy saving shows how much these scientific and political debates were determined by fears of technological as well as political path dependencies and their repercussions for the state. The paper thus proposes that the acceptance of state regulation be considered, for a better understanding of technology acceptance. The analysis of both the scientific debates and political negotiation processes together with letters from citizens to the federal government show that the criterion of distributive fairness influenced the acceptance of energy saving as a technology. Scientists, politicians, and citizens widely acknowledged that energy saving was compatible with a fair distribution of technical progress, private comfort, and economic growth, which promoted its acceptance. Moreover, their interpretation of distributive fairness also reveals an affirmative attitude towards the state and its ability to implement an acceptable energy system.

## Abstract (de)

Politiker und Regulierungsexperten sahen Mitte der 1970er Jahre das westdeutsche Energiesystem an einem Scheideweg angelangt. Sie diskutierten, ob das Energiesparen und Erneuerbare Energien oder die Kernenergie langfristig als Technologie bevorzugt werden sollten. Die Debatte um Technikakzeptanz entzündete sich nicht nur an Fragen der Wirtschaftlichkeit und den Risiken der Kernenergienutzung. Sie wurde insbesondere auch überformt von dem Streit um staatliche Regulierung, Verbraucherpartizipation und die Organisation des Energiesystems. Das Beispiel des Energiesparens zeigt, wie sehr diese wissenschaftlichen und politischen Debatten von Ängsten vor technologischen und politischen Pfadabhängigkeiten und deren Auswirkungen auf den Staat geprägt waren. Der Aufsatz schlägt hiermit vor, die Akzeptanz staatlicher Regulierung zu berücksichtigten, um ein besseres Verständnis von Technikakzeptanz zu erlangen. Die Untersuchung der wissenschaftlichen Debatten, der politischen Verhandlungsprozesse und der Briefe von Bürgern an die Bundesregierung zeigt, dass der Bewertungsmaßstab der Verteilungsgerechtigkeit die Akzeptanz des Energiesparens als Technologie beeinflusste. Wissenschaftler, Politiker und Bürger waren sich zunehmend einig, dass das Energiesparen mit einer gerechten Verteilung von technischem Fortschritt, privatem Komfort und Wirtschaftswachstum vereinbar sei, was seine Akzeptanz förderte. Ihre Interpretation von Verteilungsgerechtigkeit wies zudem eine affirmative Haltung zum Staat auf, dessen Steuerungsfähigkeit sie im Allgemeinen als notwendig erachteten, um ein akzeptables Energiesystem herbeizuführen.

## Introduction: Assessing the Impact of Energy Saving on Statehood

By the end of the 1970s, the future of the West German energy system seemed to be directly linked to the future of democracy. In 1977, the journalist Robert Jungk encapsulated such fears with his warning of an authoritarian “atomic state” (*Atomstaat*) (Jungk 1977). The “atomic state” became a widely shared reference point within the antinuclear and environmental movements, to warn of a “police state” that would become inevitable, if the risks of technical and human failures or terrorism were to be kept in check (Schüring 2015: 99–103; Seefried 2015: 487–88). Yet, a debate on technology acceptance arose also about the consequences of energy saving. During the 1970s, energy-saving measures had not only become a complementary tool for policymakers but had also been increasingly framed as the central policy alternative to the expansion of energy supply and nuclear energy (Gross 2023: 162–82; Lieb 2022: 67–81). Countering the assessment by Jungk, the physicist and nuclear energy advocate Wolf Häfele, himself concerned with a “politically acceptable, democratic solution to the energy problem,” painted an opposing horror scenario and warned of a “calorie state” (*Kalorienstaat*) to emphasize the consequences of abandoning nuclear energy and the fast breeder technology, in particular.^1^ If the Federal Republic were to predominantly pursue energy saving, renewable energies, and fossil fuels to compensate for the loss of nuclear energy, an “experiment with the well-being of all” would be conducted in a “calorie state …, in which the last calorie is guarded by the state.”^2^ Häfele did not doubt the value of energy saving, but rather its possible overall contribution to the security of the energy supply. To contain the permanent risk of energy supply shortages, “strong interventions in personal freedom and a multitude of dirigiste interventions in the economy” would become necessary, as energy consumption would need to be monitored and controlled much more closely by state authorities to prevent any unnecessary waste of energy.^3^

The ensuing debate about an energy transition and the acceptance of energy saving was no longer just one about technologies. Instead, the question of acceptance was tied up with discussions about policies and whether they indicated a change in statehood. Accompanied by a great deal of media attention, the dispute over the acceptance, opportunities, and risks of energy technologies ensued during the Enquete-Commission on the “Future of Nuclear Energy Politics” (*Zukünftige Kernenergie-Politik*), set up by the Bundestag in 1979. Comprised of scientific experts and representatives of political parties, it was tasked to formulate a recommendation on different possible “paths” in dealing with energy technologies. Already in its second internal session, party representatives from the SPD, FDP, and CDU/CSU argued about whether the West German state was on the verge of becoming an “energy shortage state,” “energy-saving state,” “calorie state,” “atomic state,” or “police state” (Altenburg 2010: 133, 181). Häfele’s warnings about energy saving found some support in both the governing and opposition parties. Their representatives in the Enquete discussed policy measures as posing a risk to acceptance: state agencies would need to survey residential buildings for proper insulation; single-family houses could be viewed as too wasteful; older buildings would need to be retrofitted by law at the cost of consumers (Vowe 1991: 248). In line with Häfele’s assumption that the sufficient exploitation of energy-saving potentials could not be achieved through other means than legal prohibitions by the state, politicians involved in the Enquete negotiated which regulatory instrument would bring about an acceptable energy system. More than anything, the legal intervention of the state in private energy consumption raised the question of acceptance, as some politicians articulated a preference for economic incentives and energy prices as policy instruments (Altenburg 2010: 181).

Assessing the sociopolitical consequences of energy saving, the Enquete Commission’s members had followed the proposal of the philosopher and physicist Klaus Michael Meyer-Abich. In 1976, he had first suggested considering the “social compatibility” (*soziale Verträglichkeit*) of energy technologies as a new qualitative criterion in political decision-making to assess their acceptability (Meyer-Abich 1976: 44–47). Like Meyer-Abich, other experts also defined energy saving as a technology that contributed to the energy system and was characterized by both behavioral and technical improvements in the use of energy.^4^ Yet, in the ensuing debate, questions of technology acceptance were not directed merely towards single energy technologies but towards systems. The management of energy systems gained importance during the 1970s and 1980s, when more and more energy sources and technologies contributed significantly to the overall supply (Graf 2024a). The connection between regulatory and technical systems was not only increasingly discussed by social scientists in terms of concepts such as “large technical systems” or “sociotechnical infrastructures” (Mayntz & Hughes 1988). As this paper will show, politicians, scientists, and citizens negotiated whether the measures used to implement and regulate energy saving as part of the larger national energy system signified an acceptable form of state action—albeit in different ways. Meanwhile, the technical functioning of technologies took a back seat in the acceptance debate on energy saving.

Starting in 1976, this paper will highlight different modes of assessing technology acceptance from above and below. I will ask how politicians, scientists, and citizens discussed the acceptance of energy saving and how they assessed it with regard to state intervention. First, I will analyze how policy experts defined and contested “social compatibility” as a new criterion of technology assessment. Secondly, I will show how “social compatibility” was taken up in political negotiations and early social science experiments with citizen participation formats. Finally, citizens’ letters to the Federal Ministry of Economics are used to show how their modes of assessing acceptance differed from those of experts and politicians. The development is traced until 1986, when the nuclear disaster in Chernobyl and the climate change debate significantly changed the momentum and political reasoning for an energy transition as well as the technology acceptance debates in West Germany (Gross 2023: 211–36). Overall, my argument is based on publications by scientists, minutes of plenary proceedings of the Bundestag, and governmental records. Looking at the case of energy saving, it expands the existing historiography on technological acceptance, risk assessments, and energy policy by analyzing the debates on sociopolitical risks in the 1970s and 1980s.

Historiography on West German energy politics has made considerable efforts to analyze the public conflict surrounding nuclear energy and its acceptance.^5^ However, the social science debate on the acceptance of energy technologies and its related contemporary concepts such as “social compatibility” has not yet been analyzed. Furthermore, recent historiography has focused on the opinions of politicians and scientific experts regarding the economic usefulness of energy technologies. In this way, Stephen Gross and Eva Oberloskamp have shown how renewable energies and energy saving have increasingly been recognized as viable “alternative” technologies while notions of ecological modernization, market liberalism, and economic policy implementation have gained importance in energy economics and politics (Gross 2023; Oberloskamp 2025). Yet, with regard to energy saving and its acceptance, the paper emphasizes that—beyond economic markets and policy instruments—a mobilizing state became the central source of hope as a driver of change in technologies and consumer behavior. The West German state figured centrally in contemporary imaginaries of implementing an acceptable and fair energy system, regardless of political conflicts over policy instruments. Ultimately, policy considerations were overshadowed by the importance of distributive fairness as a fundamental evaluation criterion for politicians, scientists, and citizens, to view energy saving as an acceptable technology.

## Conceptualizing “Socially Compatible” Energy Transitions

Technology assessment had originally been developed in the late 1960s to appraise the risks of technologies to society, the environment, and the economy (Doil 2022). During the 1970s, however, the interaction between technologies and social development came to the forefront of the debate in energy politics and scientists urged that the social and political risks of the technology be taken into account. At the German Industrial Exhibition in 1976, the philosopher and physicist Klaus Michael Meyer-Abich first suggested that “social compatibility” be considered as a new qualitative criterion in technology assessment (Meyer-Abich 1976: 44–47). A former assistant to the prominent nuclear physicist Carl Friedrich von Weizsäcker, and a professor of natural philosophy, Meyer-Abich had quickly become an influential policy expert and public advocate of energy saving during the 1970s (Oberloskamp 2025: 344–45; Seefried 2015: 343–44). While cost effectiveness and supply security had been well-established criteria by which to evaluate energy policy, he proposed, analogous to the equally new concept of environmental compatibility, that the sociopolitical consequences should also be considered in political decision-making. The starting point for Meyer-Abich’s plea for “social compatibility” as a new criterion for technology assessment was the criticism of large-scale technological systems and technocratic power, which was directed against big technology and science in the 1960s and 1970s, and in particular against nuclear energy (Augustine 2018). However, his proposal for a new instrument of technology assessment aimed, first and foremost, at increasing the efficiency, flexibility, and democratic character of the national energy system. The “technical risk of accidents” with nuclear energy seemed to him to be only a “marginal problem” (Meyer-Abich 1976: 45). The “main problem” was the “great loss of flexibility” with regards to “the state,” “later generations,” and “the individual” (ibid.: 45–46). Meyer-Abich himself referred to the US physicist Alvin Weinberg, who had warned in 1972 that energy systems based on nuclear power would have to be dependent on a stable political and social order as long as nuclear energy and its radioactive legacies necessitated management of their inherent risks (ibid: 44).

The contemporary ideal of consumer sovereignty, which was central to consumer protection and research, was at the heart of his claim to increased flexibility (Gasteiger 2010: 193–209). Meyer-Abich feared that an energy system based on nuclear energy would necessitate “compulsory connections” of consumers to the central power grid in order to amortize the high investments required, which would be incurred well in advance. This, in turn, would undo consumer choice as part of an “increasing shift from individual decision-making to an anonymous infrastructure with which one cannot identify” (Meyer-Abich 1976: 46). On the state level, he was concerned with a narrowing of opportunities for progressive political movements and that the concerns with social stability for the sake of nuclear energy would give rise to “a social conservatism that one no longer has any choice about” (ibid.: 46). All in all, nuclear energy seemed to cement in the long term the infrastructural, ecological, and sociopolitical options of the West German energy system and society. However, if there were alternatives, Meyer-Abich argued, that could achieve the same benefits of nuclear power at a lower cost to society, there would be no reason to take on its risks. Along with a growing number of energy experts calling for a more systematic inclusion of energy-saving measures in the German government’s energy policy, he believed that a more consistent focus on energy demand, instead of expanding the energy supply, would allow the West German energy system to react more flexibly to changing conditions.^6^ Meyer-Abich argued that there was no demand for energy itself, but only for the services it provides, such as heat, light, or mobility, and that these could be provided more efficiently through energy-saving measures (ibid.: 34–36, 44–46).

Meyer-Abich was not alone in his call for an energy system that conformed to the needs of society. While he had set out a preliminary framework for assessing energy technologies in terms of their sociopolitical impact, energy experts such as the US physicist Amory Lovins drew up a comprehensive scenario of a more acceptable energy system. Lovins’s concept of “soft” and “hard energy paths,” terms he first coined in 1976, became an important source of inspiration for the European and West German public debates on energy saving and renewables. It caught on, in particular, in the environmental movement and among critics of nuclear energy (Lovins 1976).^7^ In technical terms, the “hard path” stood for the approach of expanding energy supply through a centralized grid, especially through nuclear energy. In contrast, the “soft path” gave priority to the decentral use of renewable energies and energy saving and proposed using fossil fuels as transitional energy sources (Lovins 1977: 46). But the sociopolitical differences were also decisive for Lovins: “Though soft and hard paths [were] not *technically* incompatible,” they were “antagonistic in other and more important ways” (ibid.: 59). Much like Robert Jungk would argue one year later, Lovins highlighted the centralized organization of technology, capital, and regulatory power in the hands of a technocratic elite that would ultimately lead to overwhelming control over energy supply, its costs, and risk management. In contrast, the “soft path” would be “flexible, resilient, sustainable, and benign” in technological and social terms (ibid.: 38).

Within a few years, several studies in Western Europe formulated “soft energy paths” and “low energy scenarios.”^8^ While these studies remained strictly national in scope, they were part of an already longer existing, transnational intellectual tradition of thinking about the impact on society of energy sources and technologies that closed off or opened up development opportunities. Yet, before the 1970s, the notion of “high energy societies” was generally positively correlated with technical progress. The debates on the scarcity of (energy) resources and environmental degradation since the end of the 1960s had lent greater plausibility to criticism of the negative consequences of (energy) technologies, rising energy consumption, and unchecked economic growth. (Graf 2012: 75–90; Zachmann 2012: 10–25) Additionally, in the 1970s, the debate about the risks of nuclear energy flared up again with heightened intensity when fears of a “plutonium economy” were sparked by the possibility of producing and exporting nuclear weapons in new types of reactors, including the so-called fast breeder reactor (Trischler & Bud 2018: 195–200). Beyond the field of energy, Meyer-Abich and Lovins were part of a broader debate that engaged critically with technologies and featured public intellectuals such as Ernst Friedrich Schumacher, Ivan Illich, or Lewis Mumford, who had questioned narratives of technical progress and increasingly included an environmental perspective in their criticism (Meixner 1981: 332–33). Many of the “soft” energy studies adopted a social utopian perspective on technology, as they promoted ideas of simple technologies that were easy to understand and use (Oberloskamp 2025: 180–85). However, a common criterion for what constitutes “soft (energy) technologies” did not emerge: it was either assumed that the technical size and complexity increased their comprehensibility and manageability; or, on a more abstract level, the technologies’ effects on nature or society were emphasized, whereby the studies did not necessarily focus on controllability for consumers, but on risks for society as a whole. Overall, they strived for a “softer” structure of the economy, as the decentralization of small technologies would, it was felt, encourage the autonomy, creativity, solidarity, and participation of consumers in the energy system (Kitschelt 1984: 41–48).

Yet, the willing consumer often imagined at the center of the “soft path” scenarios, as part of a movement from below, was not only a romantic figment of the cultural left. There were significant socio-moral overlaps of countercultural and neoliberal ideals of subjectivity with regards to personal responsibility and its impact in consumer markets, as historians have pointed out (Möckel 2024; Bösch 2018). What’s more, much like the economist Ernst Friedrich Schumacher, who had promoted the concept of “intermediate technologies” in publications such as his influential *Small is Beautiful*, both Lovins and Meyer-Abich demonstrated with regard to energy use a contemporary search for “economic alternatives” that did not reject economic growth altogether (Möckel 2022). Likewise, they did not combine their calls for flexibility, individual technology control, and participation with countercultural ideas that necessarily promote individual autarky, which is to say, an exit from the infrastructural system of energy. They remained very much committed to the perspective of a national energy system in a conventional way and in line with contemporary regulatory debates, which was consistent with their role as established policy advisors.

While Lovins’s and Meyer-Abich’s energy paths assessments reached the environmental movement and the countercultural milieu owing to their critique of technocracy and consumerism, they made inroads with established energy expertise by framing the question of technology acceptance in terms of economic viability, security, and comfort. Meyer-Abich had originally argued in favor of qualitative economic growth and the need to arrive at least at stationary energy demand in the future, considering ecological limits to energy production and use through waste heat, aerosols, and CO2 emissions (Meyer-Abich 1973). But later, throughout the 1970s, he avoided promoting energy saving as anything other than a means to efficiency and continued economic growth. Instead, he joined in the demands for energy demand regulation to decouple the growth rates of primary energy consumption and economic production (Meyer-Abich 1979a).^9^ Much like his American counterparts, he proposed an understanding of “energy saving as a new energy source” in its own right (ibid: 17; Turnbull 2017: 433–38). Meyer-Abich argued that it could make a decisive contribution to the energy system without “restriction and renunciation of consumption,” which to him was still too much associated with energy saving (Meyer-Abich 1979a: 27). With regards to state regulation, energy saving could be done, as he claimed, “without limiting the benefits … neither at the price of dirigisme nor at the price of reducing expectations of prosperity (for example by artificially raising the energy price level)” (ibid.: 28).

Meyer-Abich’s argument in favor of energy saving reflected an emerging consensus among regulators, scientific experts, and political parties as well as antinuclear and environmental civil society groups as to how the energy system should be designed: energy savings ought to come at no costs to consumers but rather to be economically viable. In their programs and publications, these groups used the slogan of “saving energy without sacrificing comfort” (*Energiesparen ohne Komfortverzicht*) or one of its variations.^10^ This rhetoric was based on the shared value of distributive fairness, which proved to be central to the acceptance of energy savings. It was not conceptualized in terms of social justice between those who consumed more energy and those who consumed less. But it was oriented towards technical progress and economic growth, which were to be made more efficient and, as far as possible, freed from their negative side effects.

## Acceptability and Consumer Mobilization from Above

Lovins’s and Meyer-Abich’s arguments for “soft energy paths” and “social compatibility” were part of the growing calls for an energy transition. By the mid-1970s, the terms had entered into the vocabulary of governments, energy providers, and eco-socialists alike, even if these differed greatly in their definition of what a “transition” entailed (Basosi 2023). As historians Christophe Bonneuil and Jean-Baptiste Fressoz have argued, the energy transition rhetoric of the 1970s was generally underpinned by a “planning and managerial rationality” that proved to be compatible with “ecological milieus” as well (Bonneuil & Fressoz 2017: 61–62). Especially in ecological milieus, the idea of “technology developing in great radical shifts” caught on, yet at the risk of “overestimating the technological determinants” of energy transitions (ibid.).

Reflective of a planning and managerial approach to technology assessment, Meyer-Abich determined the acceptance of energy technologies not empirically, from the viewpoint of citizens, but rather intended “social compatibility” to be a “normative term” (Meyer-Abich 1979b: 40). Criticism of technocratic energy politics, therefore, did not necessarily lead to more participation of citizens in political decision-making or in determining technology acceptance. In 1979, Meyer-Abich made it clear that he deliberately defined “social compatibility” as “acceptability,” in order to distinguish his approach from the studies of empirical social research on “social acceptance,” as these “only ever result in opinions that do not necessarily have to be in harmony with the constitutional order, for example” (ibid.: 40). According to this interpretation, technology acceptance remained, first and foremost, the domain of experts and politicians. Meyer-Abich stressed that the assessment of “socially compatible” technologies was “not a technical, but a political decision” (ibid.: 39).

As historians Ute Hasenöhrl and Patrick Kupper have critically noted for the case of renewable energies, the notion of “decentralized, bottom-up, ‘soft’ energies and technologies is largely a myth, spurred by eco-legends of civil society tinkerers” (Hasenöhrl & Kupper 2021: 405). Contemporaries also already criticized the “soft path” as technologically deterministic for the far-reaching assumptions of social change (Wiesenthal 1982: 53). However, the limits to thinking in dichotomous energy systems in contemporary West German social science were also already evident in the research project that Meyer-Abich carried out together with the economist Bertram Schefold on behalf of the Federal Ministry of Research and Technology from 1980 to 1984.^11^ To assess the “social compatibility” of the West German energy system, the project’s aim was to prevent “nuclear energy as a subject [from being] left to the natural and engineering sciences” (Meyer-Abich & Schefold 1986: 35). Set up within the Federation of German Scientists (VDW), it included biologists, lawyers, economists, physicists, philosophers, psychologists, political scientists, sociologists, and historians who contributed to its internal discussions and publications.^12^

For the research group, the conceptual vagueness of “soft” and “hard” technologies became a point of contention. The economist Horst Meixner, who worked under Bertram Schefold, emphasized that scientists continued either to define “soft technologies” according to different criteria or did not define them at all. Also, he criticized the often, apodictic reasoning of “soft energy” studies, namely that small, renewable, decentralized, environmentally friendly, or more efficient energy technologies were more attuned to society’s needs. While these arguments were based rather in implicit assumptions than in proofs about their positive effects, so his argument, they also tended to neglect the negative effects, which were only highlighted in the case of “hard” technologies. Yet, energy sources and technologies tended to resist such easy categorizations along the lines of “hard” and “soft.” Heat pumps and district heating were examples of energy-saving technologies that were categorized as either “soft” or “hard,” depending on whether or not they were seen as contributing to a centralized, “hard” electricity grid (Meixner 1981: 341–46).

The participants in the research group, therefore, did not necessarily share the dichotomous thinking in terms of incompatible energy systems. In the internal research group’s discussions, they questioned whether the “handling of a specific technology … could be more decisive” than “the choice between different energy technologies,” as it seemed “possible to think of a soft nuclear energy with many small, decentralized nuclear power plants and a hard sun with a few centralized large-scale plants.”^13^ While Schefold and Meyer-Abich replied that it would be more economical to build a few large reactors which would pose no greater risk than small-scale reactors, both showed that implicit assumptions about economic actions very much drove their reasoning about the impact of “energy paths” on statehood.^14^ In the final publication of their project, they then still assumed that “energy systems” based on nuclear energy and energy saving followed their own “logic,” as did solar energy (Meyer-Abich & Schefold 1986: 163–66). In their view, the West German state, economy, and society was at a “crossroads” (ibid.: 180–83). Nuclear energy was “conceptually” geared towards “overcoming the finite nature of fossil energy resources” (ibid.: 164). The specifically nuclear “economic style” ultimately led inevitably to a “high-energy society” and to the “appropriate organization of energy use,” which would have to forcibly connect consumers to the nuclear power grid in order to be profitable (ibid.: 163). Ultimately, this inherent economic logic would also lead in the long term to the “fast breeder reactor” and the “plutonium economy” (ibid.: 163–64). In contrast, the “logic” of an “energy path” built primarily on energy saving and renewables was “less clear,” as they admitted (ibid.: 164). But, they believed, whereas nuclear energy blocked development opportunities, energy saving and solar energy would accommodate a possible shift in social values. This seemed to offer an opportunity to adapt the energy infrastructure in the future more easily to different human demands for technical progress, security, consumption, or the relationship with nature (ibid.: 86–95, 164–66).

Both Meyer-Abich and Schefold showed that it was not necessarily the distinction between large- and small-scale technologies that underlay their “energy path” scenarios. This rendered the intrinsic “logic” (*Eigensinn*) of technologies, with regard to the statehood that they assumed, ever more ambiguous and harder to grasp. After a decade of discussion in social science on “socio-technical infrastructures” and “large technical systems,” the sociologist Bernward Joerges criticized that many authors had either explicitly turned to boosters, regulators, professional networks, or consumers, as units of analysis, or had implicitly highlighted their importance as system builders. Although technologies and technical systems were central to their perspectives, these studies shared a “common feature” in their “neglect of the material-operational core of such systems” (Joerges 1988: 18). The extent to which the socio-technical infrastructures studied behaved differently from the social phenomena analyzed often remained an open question (ibid: 11–16). Instead of exhibiting the *Eigensinn* of technological development and its effects on society, Schefold’s and Meyer-Abich’s thinking, too, was strongly shaped by assumptions about the actions of politicians, companies, and consumers that were present in contemporary critiques of technocracy, consumerism, and materialist values.

The opportunity that an energy system built around energy saving and solar energy seemed to represent for democratic culture, social value change, sustainability, and good governance did not necessarily follow from the technology’s size and decentralization. It very much hinged in these studies on the assumed willingness of consumers to participate and on the state’s capacity to implement policies. When it came to making consumers active participants in energy saving, there were clear differences with regard to what constituted acceptable and necessary policies. Whereas Lovins associated “soft” regulation of energy demand largely with economic incentives and market-based instruments, Meyer-Abich and Schefold assumed that the state would be able to implement and finance the energy transition through legal efficiency standards and subsidies (Turnbull 2017: 365–67). What distinguished Meyer-Abich from Lovins as well as from most West German ordoliberal energy economists, was his skepticism as to whether consumers would react sufficiently to economic incentives, such as rising energy prices or taxes on energy demand, to exploit existing energy-saving potentials. He argued instead that energy savings would come down to technical progress that the state needed to accelerate through a variety of regulatory instruments, such as higher legal efficiency standards for buildings and heating systems, and massive subsidies. Together with the economist Hans-Joachim Luhmann, Meyer-Abich recommended that distributive fairness should be taken into account, as economic incentives alone would lead to an extremely unequal burden on consumers (Meyer-Abich 1979a: 79–87, 228–35). These assumptions about the governability of energy consumers differentiated them from critics such as Wolf Häfele, who did not doubt that economically and technically feasible energy-saving potentials existed. Yet, Häfele doubted whether consumers would participate sufficiently to foster an energy system largely eschewing nuclear energy. He instead made an argument for increased centralization of the energy system: “Very strong and extreme energy-saving measures are only effective if they reach every leaf in the widely ramified tree of energy use. … Measures on the side of centralized energy supply, on the other hand, are easier to oversee and easier to implement.”^15^

In contrast to the scenarios of seemingly incompatible energy technologies, the question of how to mobilize consumers to initiate an energy transition did not divide experts in West Germany in line with fundamental distinctions such as unregulated markets or political planning. Instead, differences over state regulation were rather gradual than fundamental, as all the proposed measures employed a mixture of economic, informational, and legal policy instruments. The ecological and antinuclear wing of the SPD, where politicians pushed for higher subsidies and efficiency standards, likewise shared Meyer-Abich’s skepticism concerning the energy price mechanism (Gross 2023: 173–81; Lieb 2022: 67–81). In contrast, the Federal Ministry of Economics, which was generally closer to ordoliberal economists, showed greater confidence in energy prices, but by no means relied solely on market processes and economic incentives to persuade consumers to save energy. An expression of this was the Energy Saving Act (*Energieeinsparungsgesetz*), which the German government passed in 1976. It stipulated several regulations for thermal insulation standards in new buildings as well as the inspection and maintenance of heating systems. In addition, subsidy programs aimed to encourage consumers to invest in energy-efficient household infrastructure while taxes on heating oil and mandatory consumption-based heating bills were intended to create additional economic incentives (Meyer-Renschhausen 1981: 127–205).

## The Adoption of “Social Compatibility” in Politics and a Turn Towards Empirical Consumer Research and Citizen Participation in Social Science

In 1980, the influential “Energie-Wende” study by the Öko-Institut (Eco-Institute), which had a major impact on the thinking of environmental and antinuclear groups and think-tanks at the time, also adopted and adapted the criterion of “social compatibility.”^16^ Its authors proposed considering the freedom of action of institutions, of citizens, the government, and energy markets as well as the versatility of energy supply for energy technologies, yet emphasized the participation of consumers more explicitly than Meyer-Abich had (Krause et al. 1980: 207–10, 219–22). Beyond environmental think-tanks, the Enquete Commission, which was tasked with formulating a recommendation on “Future Nuclear Energy Policy,” showed that political parties in the late 1970s were approaching a broader consensus on the inclusion of qualitative criteria for technology acceptance in political decision-making. While it was a priority point in the discussion on quantitative energy prognoses, the scientific experts and representatives of the political parties at the Enquete now pushed for the inclusion of value judgements to determine the level of acceptable costs and benefits in energy policies. Following the suggestion of Meyer-Abich, the members of the Enquete included “social compatibility” as a criterion for evaluating energy technologies. In their first report to the Bundestag, they defined it as compliance with the constitutional order, the revisability of political decisions, the freedom to shape one’s own life, and the flexibility of future generations.^17^

The scientific experts and politicians of the Enquete formulated a shaky compromise that outlined four different “energy paths” up to the year 2030 that attached very different levels of importance to nuclear energy and energy saving. In the end, all members considered all four paths to be feasible, yet not necessarily desirable. This compromise reflected the broader contemporary consensus on distributive fairness among energy experts. The decisive factor for acceptance of all paths was the premise that they should be economically feasible given the existing technologies, and not compromise the comfort of consumers. There was also a fundamental consensus that energy-saving measures were needed for all paths, which contained different levels of savings targets. The paths differed in terms of the economic growth rates assumed necessary to support economic structural change and combinations of energy technologies. Among its recommendations, the Enquete also formulated a comprehensive catalogue of 62 energy-saving measures. On this basis, the Commission proposed that a final decision on the long-term use of nuclear energy, and in particular of the fast breeder technology, should not be made before 1990. Until then, nuclear power plants should only be built as required if there were no other options for covering energy demand through energy saving or renewable energies (Altenburg 2010: 37–44, 179–85). With regards to governmental energy-saving measures, the proposal that state authorities should examine the compliance of residential buildings with existing building codes for proper thermal insulation, that only multifamily apartment buildings should receive state funding, and that also older buildings needed to comply with legal standards were heated talking points during the Enquete’s deliberations (Vowe 1991: 248).

Ultimately, the Enquete’s CDU and CSU members gave in to mounting party pressure over the Enquete’s recommendations and declared at the last possible minute that they were no longer ready to support them. Yet, the division exhibited the similarities rather than the differences in their assessment of which energy-saving strategies were acceptable. When the CDU and CSU politicians presented their own, modified version of the Enquete’s catalogue of energy-saving measures, it appeared that both catalogues were largely congruent. What caused unrest in the CDU und CSU were the proposals to commission state authorities to monitor buildings and heating systems for compliance with legal standards. These differences on policy reflected the concerns over statehood that the CDU and CSU politicians politicized in the subsequent parliamentary debate. CDU politician Lutz Stavenhagen criticized “the state’s interference in individual consumer decisions” and the “require[d] state coercion” as proposed in the Enquete’s recommendations.^18^ Rhetorically drawing a comparison with a totalitarian state, he contended that the “path without nuclear energy … described by its disciples as ‘soft,’ turns out on closer inspection to be a path full of state regulation, a total energy-saving state.”^19^ Yet, despite this rhetoric and the differences in the two energy saving catalogues, a more fundamental consensus in technology acceptance parameters came to light. Stavenhagen made clear that public acceptance should be central to the political deliberation of energy-saving measures by maintaining that “energy-saving measures must also be economically justifiable and socially acceptable.”^20^ Ultimately, he justified his party’s position by claiming that the Enquete’s recommendation on energy saving “is rightly not recognized by the consumer.”^21^ Although Stavenhagen did not clarify on what empirical basis he arrived at his assessment, he illustrated at least the rhetorical significance of consumer acceptance in the political debate.

In the early 1980s, social scientists increasingly turned to energy politics, as policy experts, and started to investigate energy-saving strategies in the light of the public energy transition debates (Graf 2024b: 305–16). Sociologists Bernward Joerges and Marvin Olsen, among others, spearheaded a broad-based investigation into consumer research from below at the International Institute for Environment and Society (IIUG) at the Berlin Social Science Center (WZB). In particular, they expressed a strong dissatisfaction with “technical fixes” in energy policy. They argued that viable technologies for energy saving had long been available to a sufficient extent. However, far too little was understood about how social contexts, socioeconomic status, experiences, values, and perceptions shaped consumer behavior (Joerges & Olsen 1979: 9–15). This problematization of consumer participation differed from the broad demoscopic acceptance research, which had only asked about citizens’ self-reported attitudes towards energy technologies and towards nuclear energy in particular. Accordingly, governmental attempts to influence public opinion on energy had conceived of technology acceptance as a deficit in information, knowledge, and trust in institutions. Programs such as the “citizens’ dialog on nuclear energy” (*Bürgerdialog Kernenergie)* in the late 1970s had assigned a passive role to citizens within a transmitter-receiver model of pedagogic science (Meyer & Oertel 2024: 44–46). In contrast, at the turn to the 1980s, social scientists attempted to better understand what shaped citizens’ behavior and perceptions in the first place. It was their goal to improve both governmental regulation techniques and consumers’ participation in energy saving. The very starting point for such research was studies that had found a discrepancy between self-reported approval of energy saving and actual behavior in daily life (Graf 2024b: 305–16).

Ministerial officials at the Federal Ministry of Research and Technology used this criticism within the scientific community as an additional argument for commissioning another research project to investigate the “social compatibility” of the energy system. Ministry officials had proved to be “skeptical, and in most cases even dismissive” of Meyer-Abich and Schefolds’ research project, as they feared it could worsen the public’s perception of nuclear energy.^22^ Led by sociologist Ortwin Renn, who had previously proved to be critical of the “soft energy” concept, a research group at the Nuclear Research Facility in Jülich (Kernforschungsanlage Jülich) pursued the first national citizens’ report (*Bürgergutachten*) in West German energy politics between 1982 and 1985 (Oesterwind et al. 1980; Renn et al. 1985). Differentiating themselves from the “normative approach” of Meyer-Abich and Schefold, they aimed instead to determine the “social compatibility” of energy scenarios based on the “subjective values of the population” (Albrecht et al. 1986: 36). This did not aim at citizen participation in political decision-making, but in policy advice. Of course, this followed governmental demands to find scientific solutions to antinuclear protests and led, ultimately, to “controlled citizen consultation [rather] than direct citizen democracy,” as political scientist Thomas Saretzki would later comment (Saretzki 2005: 647–48).

The citizens’ report by the Jülich research group, therefore, remained very much mediated by scientists. Although it reflected a heightened interest in dialogue and not just in the mere dissemination of information to citizens, it would remain the only citizens’ report at the national level in the 1980s, and it did not have a tangible impact on negotiations in parliament, public discourse, or in the federal ministries. With regards to the acceptance of energy saving, it exhibited significant overlaps with earlier scientific and political assessments. As Renn and his research group highlighted from the interviews they conducted among participants, “fears of a development into an ‘atomic state’ or ‘calorie state’ had found little resonance” (Renn et al. 1985: 147). Rather, participants affirmed the necessity of government measures. Even those in favor of nuclear energy were “relatively indifferent to fears that the energy-saving state could lead to similar restrictions on personal freedom as the ‘atomic state’” (ibid.: 155). In the final report, citizens concluded that energy-saving measures were just as important in their own recommendations as nuclear energy and other energy sources (Dienel & Garbe 1985: 144). What’s more, in the West German debate, neither the “calorie state” nor its semantic variants established themselves as political “battle terms” in public usage, not even in the public relations work of the energy industry during the 1980s and beyond (Jung 1994: 110, 190).

In terms of specific measures, the citizens’ report highlighted the preference of participants. They were tasked with selecting energy-saving measures that they considered acceptable and effective to achieve the different energy savings targets that the Enquete had assumed for its four “energy paths” until 2030. Overall, the citizens’ recommendations largely corresponded to the compromise that the Enquete had originally found with its energy savings catalogue. However, all the “energy paths” formulated by the citizens included stricter statutory efficiency requirements for new buildings and heating systems, state subsidies for retrofitting existing buildings and heating systems, and consumer information measures. Citizens with a preference for paths with higher savings rates also showed a greater willingness to sacrifice comfort to promote energy saving, while such positions had not gained any traction on the political stage. Nevertheless, monitoring of the legal standards and savings efforts by consumers proved to be just as controversial as on the political stage and the willingness to accept reductions in personal standards of living found only little support (Dienel & Garbe 1985: 147–55).

## Credibility, Fairness, and Economic Benefits as Criteria of Distributive Fairness. Citizens’ Letters to the Government

In addition to the assessments of politicians and scientific experts, direct communication between citizens and the government can be used to gauge the acceptance of energy saving. Letters from citizens to the government at times influenced negotiations on regulatory projects. Among other policies, the Federal Ministry of Economics defended in 1979 the legal requirement for consumption-based heating cost billing as well as an increase in efficiency standards for buildings, heating, and hot water use with “the overwhelming desire of tenants” that it had deduced from citizens’ letters.^23^ From a more theoretical point of view, the letters provide insights into the relationship of politicians, ministry officials, and citizens (Gatzka 2024: 189–95). Accordingly, they allow one to analyze the responsibility that politicians, ministerial officials, and citizens ascribed to the state and to themselves in relation to political-economic processes such as energy saving. Despite the impression “that this letter could not achieve anything,” as citizens sometimes asserted, the fact of writing nevertheless expressed a sense of responsibility and a need to be heard.^24^ Furthermore, the letters analyzed below were not only letters of protests, but, in most cases, initiatives appealing to the government to regulate energy consumption more effectively or fairly. In this respect, these letters were an expression of basic trust in the state’s ability to regulate as well as a demand that it should do so in accordance with the respective writer’s wishes. Today, 17 volumes of documents—stored in the Federal Archives and each containing around 30 to 50 letters on the subject of energy saving—still testify to the intensive exchange that took place between citizens and the government between 1976 and 1986.^25^ Although the ministries did not keep statistics on the number of letters received, ministry officials repeatedly referred to their great quantity: “every day, we receive a large number of letters with suggestions and ideas for saving energy.”^26^

Above all, these interactions between citizens and government representatives not only help to substantiate or challenge the findings made in the scientific and political debates on the acceptance of energy saving. They also reveal different modes by which citizens judged their acceptance. Although citizens also discussed the acceptance of energy saving in relation to the issue of state regulation, they used a different set of assessment criteria. They primarily judged government measures according to their credibility, fairness, and benefits. Historians have already emphasized the importance of sociocultural factors for the case of nuclear energy. They have pointed to the importance of values in the formation of trust in public institutions and energy companies to explain the discrepancy in very similar benefits and risk perceptions with diverging patterns of acceptance in several European countries during the 1970s and 1980s (Espluga et al. 2021: 164–65). Similarly, through letters, citizens negotiated whether they should have confidence in the credibility, fairness, and benefits of government energy-saving measures. An equal distribution of costs, benefits, and responsibility in the saving of energy was more crucial than the notion that technologies would substantially alter the social and political fabric. Citizens tended to focus on the individual functioning of energy-saving measures to assess their acceptability, and not on their compatibility with a market-based or state-centered energy policy, as the public regulatory debates would suggest as the central distinction.

Notions such as the “calorie state” were equally not important to citizens when they spoke out in letters to the ministry in favor of or against government energy-saving measures, confirming the results of the citizens’ report by the Nuclear Research Facility in Jülich. Nor did citizens perceive energy saving and nuclear energy as incommensurable technologies. Moreover, the letters mentioned to very different extents the regulatory, information, and financial policy energy-saving measures enacted by the federal government up to the beginning of the 1980s. In particular, the Energy Saving Act, passed in 1976, and the subsequently enacted legal requirements for buildings and heating systems hardly aroused the citizens’ displeasure. Citizens, instead, paid much closer attention to the government’s public relations and energy-savings campaigns, which often provoked opposition. That the federal government appealed to citizens to save energy but seemed to remain inactive itself triggered a flood of letters: “I don’t understand how you can bring out brochures about saving energy when you don’t take any action,” as one citizen wrote.^27^ Citizens even reminded the federal ministries of their own rhetoric, in writing that “energy saving” was “our best source of energy,” citing directly from information brochures.^28^ Along these lines, citizens complained frequently about the inconsistency of the governmental energy-saving policies. For them, it seemed contradictory to their own efforts to save energy if barriers to saving were not removed, or significant opportunities to save were not exploited or even considered by the government. One citizen referred to building regulations that prevented the installation of solar systems in his home and criticized that “all appeals to save energy … are paradoxical if the corresponding regulations, ordinances, or laws that prevent real energy saving are not immediately revoked!”^29^

Citizens emphasized the inconsistency of energy policy often with regard to the actions of public institutions, utilities, businesses, and of other consumers, which they perceived as an inappropriate luxury in times of resource scarcity: car racing, neon signs, street lighting, or politicians’ air travel were frequent targets of such criticism.^30^ In line with the high visibility and symbolic importance of traffic, an official of the Federal Chancellery had repeatedly warned Helmut Schmidt in 1979, ahead of the upcoming parliamentary election, that the mobilization of consumers was dependent on the personal behavior of members of the government. He recommended “fewer campaign appearances rather than lots of helicopters and hurried Mercedes convoys. Traffic is the most important energy experience for the average citizen.”^31^ Accordingly, a citizen argued in a letter that the high energy consumption of “car racing … and rally driving” made her own efforts seem futile.^32^ Still, citizens proved to be heavily influenced by public debates. During the second oil crisis, there was a widespread debate in public media about further energy-saving measures by the state, and citizens measured the proposals against their memories of the first oil crisis, asking why a reintroduction of the temporary Sunday driving bans was not reconsidered, or why speed limits weren’t discussed.^33^ Attaching newspaper articles and publications to their letters, they tried to bolster their arguments and show that the government’s public statements conflicted with media reports.^34^

Officials in the Ministry of Economics tried to defend the ministry’s position on energy policy against both the notion of “state coercion” and the increasing calls for stronger state intervention. At times, they referred to an ideal of economic rationality and responsibility, when responding to letters, claiming that “the economical use of energy resources is primarily the responsibility of the economy and consumers” and that “only the market or the market price” should primarily guide the actions of economic entities, as is regularly the case.^35^ Yet, while positing that “experience shows that price as a scarcity gauge—combined with broad public education—is still the best means of bringing about energy-conscious consumer behavior,” ministerial officials also tried to dispel the impression of the state’s inaction.^36^ Time and again, they pleaded “that the Federal Government is by no means restricting itself to appeals for measures” and pointed to the varied saving measures as well as the mix of regulatory instruments that it used to this end.^37^ Against further and stricter legal efficiency standards, they referred to the “principle of economic viability,” which the ministry had encoded in the Energy Savings Act in 1976 as its assessment of fairness and acceptability.^38^ Only “what is economically justifiable,” which is to say, measures that would amortize within their usual lifespan, could be prescribed by law.^39^

Yet, the feeling of state coercion was not necessarily shared by citizens when it came to further economic or legal measures. Citizens often felt that “the major consumers” were located in major industries and called on the federal government to make sure that they and their customers acted more responsibly by “switching to less energy-intensive products.”^40^ The writers of letters, therefore, not only directed their criticism towards the state or companies but also towards other energy consumers. Personal efficacy—or rather the lack thereof—was a central topic in the letters and writers felt that it was often hindered by a general lack of awareness, as “saving energy … still seems to be an avoidable evil for most people. … [E]ven the most consistent individual savers can do little to change this situation as long as large consumers and the broad mass of the population continue to throw energy out the window, so to speak.”^41^ As such, letters repeatedly questioned the distributive fairness and effectiveness of energy policy. For one citizen, the way in which Federal Minister of Economics Otto Graf Lambsdorff described the rise in energy prices caused by the second oil crisis as a means of energy-saving policy in an interview in *Brigitte* magazine was “an impertinence.”^42^ She sent to the Ministry of Economics a letter that had been printed in the consumer magazine *Brigitte* and signed by multiple readers: they rejected the assumption that (to quote Lambsdorff), “the best motivation for saving energy … is, of course, the price.”^43^ This is “no contribution to combating the current price situation for heating oil,” as it burdens “families with lower and medium incomes.”^44^ Above all, and despite the critiques, letters such as these expressed a trust in the state’s capacity to remedy shortcomings and to ensure a fair distribution of the burden of energy saving, but not necessarily a trust in the current government’s ability.

Quite in line with the stance of the Ministry of Economics, however, citizens also often shared the conviction that higher energy prices would have a strong effect on their behavior.^45^ Even after the rise in energy prices following the second oil crisis, calls for price policy measures reached the federal government. Proposals to reflect the scarcity of finite fossil fuels in energy prices through taxes were largely in line with the recommendations of a growing number of economic experts, particularly from the “ecological modernization” camp (Gross 2023: 183–210). One psychologist and economist by profession wrote to the Ministry of Economics that he supported the idea of raising energy prices through “VAT increases … for heating energy” and said that “the psychological situation is more favorable than ever before.”^46^ After the nuclear accident in Harrisburg and the second oil crisis, “one can hope for the population’s understanding for such a measure.”^47^ In view of the “transition to the post-oil age,” another citizen believed that “appeals for moderation alone … will not be enough” and therefore suggested “an increasing tax burden on oil products”: “We are all … used to wasting energy. … I hope, in the interests of the generation after next, that we alive today will soon realize that our luxury consumption cannot be justified.”^48^

However, the frequent appeal in letters for all energy consumers to assume shared responsibility, which was characteristic of the government’s rhetoric as well, was also reinforced by citizens’ understanding that increased energy savings would necessarily entail a sacrifice of personal comfort—as social science studies, evaluating government energy-saving measures, found. The message from the German government and many experts, that there were sufficient ways to save energy without any reduction in living standards, often did not reach consumers (Mettler-Meibom & Wichmann 1982: 75–78). In particular, the Chernobyl nuclear disaster strengthened once again this type of petition. Citizens emphasized that they were prepared to “make sacrifices (for instance, less electricity consumption …)” or “do laundry and cook food at night,” outside the power grid’s peak hours, if the government would phase out nuclear energy.^49^

## Conclusion: the State at the Center of an Acceptable Energy System

This paper has suggested that the acceptance of energy-saving technologies was closely tied to questions of acceptance of policy measures during the 1970s and 1980s. In parallel to a comprehensive public debate on the technical risks of large-scale technologies, political risks to democracy and economic policies were also identified and negotiated. The emerging debate about the “social compatibility” of energy saving pushed questions concerning the technical functionality of saving measures and their integration into everyday social contexts virtually into the background. Contrary to criticism directed at early assessments of technology acceptance, citizens were neither conceived of as a passive mass of technology supporters or opponents, nor did citizens themselves exhibit NIMBYism. Citizens based their acceptance—as their letters demonstrated—upon their perceived personal efficacy as well as upon the distributive fairness of, and their trust in, governmental energy policy programs. As I have shown, citizens often called for the federal government to take stronger regulatory action and accepted the basic assumption that everyone should contribute to saving energy.

This affirmative attitude towards the state and its ability to implement an acceptable energy system was quite in contrast to larger, contemporary intellectual debates. The state’s capacity to facilitate social, technical, and economic progress had been a contentious talking point in the debate about a “legitimacy crisis” of the state and the “ungovernability” of society (Hacke 2008; Metzler 2005: 404–11). In the debate on the acceptance of energy technology and energy saving, the results of this paper point to a development that runs counter to these notions. Quite in line with recent findings by historians for other policy areas, this paper shows that intellectual, political, and media debates often differed greatly from citizens’ perceptions (Fabian 2016: 432; Gotto 2018: 114–15). Furthermore, we can observe that even scientific experts and intellectuals critical of technocratic power structures did not doubt the state’s capacity to regulate, but rather envisioned it as a necessary actor when it came to mobilizing consumers to tap into their energy-saving potentials. Only the specific policy instruments of state regulation to bring about technical and behavioral change were controversial. With policy instruments encompassing legal efficiency standards, economic incentives, and consumer information, the negotiation processes showed that neoliberal concepts of economic policy had only limited influence during the 1980s.

More pertinently, this paper has proposed distributive fairness as an analytical lens through which to better understand the contemporary valuation of the state as an actor of change in implementing a more acceptable energy system. Considering this, negotiations on energy saving policy remained very much dominated by a small group of energy experts in science and politics, and technology acceptance was largely defined and decided by struggles over their respective interpretative authority. In addition to an increased interest in citizens’ value judgements and collective participation in policy advice, this shift in acceptance research often remained very much focused on citizens as consumers, so as to improve their participation in energy markets and the regulation of energy demand. These developments also characterize a “democracy of negotiation,” as highlighted for the nuclear energy debate (Uekötter 2022: 293–305), but they did not necessarily entail a shift towards more participation by citizens in political decision-making.

## Acknowledgements

I would like to thank the peer-reviewers and the editors as well as Laura Kaiser, Simon Specht, Fabian Zimmer, Tabea Nasaroff, Rüdiger Graf, and Christopher Neumaier for their comments and suggestions that substantially improved the manuscript.

## Footnotes

1 Bericht der Enquete-Kommission „Zukünftige Kernenergie-Politik“ über den Stand der Arbeit und die Ergebnisse gemäß Beschluß des Deutschen Bundestages, in: Deutscher Bundestag, 8. Wahlperiode, Drucksache 8/4341, 27.06.1980, p. 81.

2 Ibid., p. 79.

3 Ibid.

4 Although energy savings could also refer to a reduction in individual living standards, contemporary policy studies and government measures to save energy were primarily concerned with the “rational use of energy.” This definition also went beyond the notion of efficiency improvements. See, for example, Bauerschmidt (1985: 86–95) and Schaefer (1980), who defined the “rational use of energy” as the avoidance of unnecessary consumption (e.g. overheating of rooms without additional comfort gains), the reduction of specific energy requirements through engineering, the improvement of the efficiency of technical systems and machines, energy recovery, and the use of renewable energy sources.

5 For examples of recent contributions, see Altenburg (2010), Augustine (2018), Espluga et al. (2021), Schüring (2015), Uekötter (2022).

6 For examples of early energy saving studies, see Schmitt et al. (1976), Wahl (1977).

7 See Basosi (2023: 111–12), Graf (2014: 376–77), Hesse (2016: 140–42).

8 See, for example, Leach (1979), Krause et al. (1980), Lönnroth et al. (1980).

9 On the West German decoupling-debate, see Gross (2017).

10 See, for example, Bundesministerium für Raumordnung, Bauwesen und Städtebau (1977: 6–7), Bundesverband Bürgerinitiativen Umweltschutz (BBU) (1976: 1), Informationszentrale der Elektrizitätswirtschaft (1978: 6), Ruske & Teufel (1980: 17).

11 On Schefold, see also Oberloskamp (2025: 480–82).

12 For the members and the organization of the research project, see Meyer-Abich & Schefold (1986: 199–208).

13 Protokoll der Studiengruppensitzung am 07.11.1980 in Berlin, Wissenschaftszentrum Berlin für Sozialforschung, Bundesarchiv, BArch, B 196/52961.

14 Ibid.

15 Bericht der Enquete-Kommission „Zukünftige Kernenergie-Politik“ über den Stand der Arbeit und die Ergebnisse gemäß Beschluß des Deutschen Bundestages, in: Deutscher Bundestag, 8. Wahlperiode, Drucksache 8/4341, 27.06.1980, p. 79.

16 On the importance of the study in energy and environmental politics, see Oberloskamp (2025: 182–84).

17 See also Bericht der Enquete-Kommission „Zukünftige Kernenergie-Politik“ über den Stand der Arbeit und die Ergebnisse gemäß Beschluß des Deutschen Bundestages, in: Deutscher Bundestag, 8. Wahlperiode, Drucksache 8/4341, 27.06.1980, p. 13.

18 Lutz Stavenhagen, Beitrag in der Beratung des Berichts über den Stand der Arbeit und die Ergebnisse der Enquete-Kommission „Zukünftige Kernenergie Politik“, in: Deutscher Bundestag (ed.), Stenographischer Bericht, 229. Sitzung (= Plenarprotokoll 8/229), 03.07.1980, p. 18625–626.

19 Ibid.

20 Ibid.

21 Ibid.

22 311, Betr.: Schriftliche Frage eines Mitglieds des Deutschen Bundestages, Bonn, den 12.10.1981, BArch, B 196/52961. See also the comments of Meyer-Abich & Schefold (1986: 199).

23 Der Leiter der Abteilung III, Herrn PSt Grüner, StS Dr. von Würzen, Betr.: Sprechzettel für die Sitzung des Bundeskabinetts am 15.08.1979, Bonn, den 14.08.1979, BArch, B 102/338198.

24 Johannes G. an Bundeswirtschaftsminister Martin Bangemann, 07.05.1986, BArch, B 102/349273.

25 For these records, see BArch, B 102/200795 to 200800, 260338 to 260343, 278529, 289619, 303079, 349273.

26 III A 6, Herrn Rudolf P., Betr.: Energieeinsparung, 20.02.1980, BArch, B 102/303079.

27 Jürgen R. an das Bundesministerium für Wirtschaft, 28.03.1980, BArch, B 102/289619.

28 Andrea K., Herrn Bundeswirtschaftsminister, Betr.: Kernreaktorkatastrophe in Tschernobyl, 21.05.1986, BArch, B 102/349273.

29 Hans-Paul P. an den Herrn Bundesminister für Wirtschaft, Betr.: Energiesparen, 03.03.1980, BArch, B 102/289619.

30 See, for example, Initiative Energieeinsparung, An den Bundesminister für Wirtschaft und Technik, 03.03.1980, BArch, B 102/289619; Gerhard J., Herrn Graf v. Lambsdorff, Betr.: Bau einer Rennsportanlage, 20.04.1980, BArch, B 102/289619; Walter S., An den Herrn Wirtschaftsminister Dr. Friedrichs, 08.12.1975, BArch, B 102/200795.

31 H. V. Ziegler, Prognose der weiteren Entwicklung in der innenpolitischen Energiediskussion, Bonn, den 24.08.1979, BArch, B 136/11016.

32 Ralf D., An Herrn Graf von Lambsdorff, 11.06.1979, BArch, B 102/200798.

33 See, as examples, H. Becker an Herrn Graf Lampsdorf [*sic*], Betr.: Energiesparen, bei der Behörde, im Haushalt und Auto, Bezug: Die Meinung des Wirtschaftsministers in den Tageszeitg., 23.04.1980, BArch, B 102/289619; S. Dieregsweiler/P. Comanns, 23.04.1980, BArch, B 102/289619.

34 See Karl S., [Mai] 1979, BArch, B 102/200798.

35 IV C 7, Bundesministerium für Wirtschaft an Herrn Klaus J., 13.05.1980, BArch, B 102/289619.

36 Referat III D 1, Bundesministerium für Wirtschaft, an Herrn Walter H., 29.05.1979, BArch, B 102/200798.

37 Ibid.

38 III A 6, Frau M. S., Herrn H. S., 16.05.1983, BArch, B 102/303079.

39 Ibid.

40 Referat IV C 7, Bundesministerium für Wirtschaft an Herrn Klaus J., 13.05.1980, BArch, B 102/289619.

41 Initiative Energieeinsparung, An den Bundesminister für Wirtschaft und Technik, 03.03.1980, BArch, B 102/289619.

42 Anni M., an das Bundeswirtschaftsministerium, Herrn Wirtschaftsminister Graf Lambsdorff, 21.06.1979, BArch, B 102/200798.

43 Ibid.

44 Ibid.

45 Ingrid S., Bundesministerium für Wirtschaft, Betreff: Beschwerde gegen Verschwendung von Benzin und Energie, 09.02.1983, BArch, B 102/303079.

46 Viktor M., An die Herren Bundesminister für Finanzen, Verkehr, Wirtschaft, Inflationsneutrale, voraussichtlich sehr erhebliche Einsparung von Energie in privaten Haushalten in Verbindung mit Maßnahmen zur Herleitung der Steuerreform, Augsburg, den 06.04.1979, BArch, B 102/200798.

47 Ibid.

48 Walter H., Bundesministerium für Wirtschaft, 08.06.1979, BArch, B 102/200798.

49 Nina M., Freiburg 08.05.1986, BArch, B 102/348273; Beate D., Thomas D., An alle Regierungen der Bundesrepublik Deutschland, Tschernobyl und die Konsequenzen für das Leben mit Atomanlagen, Mai 1986, BArch, B 102/348273.

## References

[CR1] Albrecht, Gabriele, Ortwin Renn, Ulrich Kotte, Hans Peter Peters, and Hans U. Stegelmann 1986. Sozialverträgliche Energieversorgung. Ein empirischer Ansatz zur Analyse von Bürgerpräferenzen in der Energiepolitik. *Aus Politik und Zeitgeschichte* (36/32): 35–48.

[CR2] Altenburg, Cornelia 2010. *Kernenergie und Politikberatung. Die Vermessung einer Kontroverse*. Wiesbaden: VS Verlag für Sozialwissenschaften.

[CR3] Augustine, Dolores L. 2018. *Taking on Technocracy. Nuclear Power in Germany, 1945 to the Present*. New York, NY: Berghahn Books.

[CR4] Basosi, Duccio 2023. The Decade of the “Energy Transition.” A Critical Review of the Global Energy Debates of the 1970s. In: Stephen G. Gross and Andrew Needham (eds.). *New Energies. A History of Energy Transitions in Europe and North America*. Pittsburgh: University of Pittsburgh Press: 107–18.

[CR5] Bauerschmidt, Rolf 1985. *Kernenergie oder Sonnenenergie*. Munich: Beck.

[CR6] Bonneuil, Christophe and Jean-Baptiste Fressoz 2017. *The Shock of the Anthropocene. The Earth, History and Us*. London: Verso.

[CR7] Bösch, Frank 2018. Krisenkinder. Neoliberale, die Grünen und der Wandel des Politischen in den 1970er und 1980er Jahren. In: Frank Bösch, Thomas Hertfelder, and Gabriele Metzler (eds.). *Grenzen des Neoliberalismus. Der Wandel des Liberalismus im späten 20. Jahrhundert*. Stuttgart: Franz Steiner Verlag: 39–60.

[CR8] Bundesministerium für Raumordnung, Bauwesen und Städtebau 1977. *Energiesparbuch für das Eigenheim. Eine Anleitung zu Verbesserungen an Haus und Heizung*. Bonn, Berlin.

[CR9] Bundesverband Bürgerinitiativen Umweltschutz (BBU) 1976. *Energie und Haushalt*. Karlsruhe: BBU.

[CR10] Dienel, Peter C. and Detlef Garbe (eds.) 1985. *Zukünftige Energiepolitik. Ein Bürgergutachten*. Munich: High-Tech-Verlag.

[CR11] Doil, Lukas 2022. Zukunftspolitik im Technozän. Der Technikfolgendiskurs in den 1970er Jahren. *Kulturwissenschaftliche Zeitschrift* (7/1): 88–106.

[CR13] Espluga, Josep, Wilfried Konrad, Ann Enander, Beatriz Medina, Ana Prades, and Pieter Cools 2021. Risky or Beneficial? Exploring Perceptions of Nuclear Energy over Time in a Cross-Country Perspective. In: Arne Kaijser, Markku Lehtonen, Maria Mar Del Rubio, and Jan-Henrik Meyer (eds.). *Engaging the Atom. The History of Nuclear Energy and Society in Europe from the 1950s to the Present*. First ed. Morgantown: West Virginia University Press: 147–69.

[CR14] Fabian, Sina 2016. *Boom in der Krise. Konsum, Tourismus, Autofahren in Westdeutschland und Großbritannien 1970–1990*. Göttingen: Wallstein Verlag.

[CR15] Gasteiger, Nepomuk 2010. *Der Konsument. Verbraucherbilder in Werbung, Konsumkritik und Verbraucherschutz. 1945–1989*. Frankfurt am Main, New York: Campus-Verlag.

[CR16] Gatzka, Claudia C. 2024. Feedback aus der Peripherie der Demokratie. Bürgerbriefe und politische Repräsentation in der Bundesrepublik (1949–1980). In: Ernst Wolfgang Becker and Frank Bösch (eds.). *Partizipation per Post. Bürgerbriefe an Politiker in Diktatur und Demokratie*. Stuttgart: Franz Steiner Verlag: 187–215.

[CR17] Gotto, Bernhard 2018. *Enttäuschung in der Demokratie. Erfahrung und Deutung von politischem Engagement in der Bundesrepublik Deutschland während der 1970er und 1980er Jahre*. Berlin, Boston: De Gruyter Oldenbourg.

[CR18] Graf, Rüdiger 2012. Von der Energievergessenheit zur theoretischen Metonymie. Energie als Medium der Gesellschaftsbeschreibung im 20. Jahrhundert. In: Hendrik Ehrhardt and Thomas Kroll (eds.). *Energie in der modernen Gesellschaft. Zeithistorische Perspektiven*. Göttingen: Vandenhoeck & Ruprecht: 73–92.

[CR19] Graf, Rüdiger 2014. *Öl und Souveränität. Petroknowledge und Energiepolitik in den USA und Westeuropa in den 1970er Jahren*. Berlin: De Gruyter Oldenbourg.

[CR20] Graf, Rüdiger 2024a. Energie. In: Ernst Müller, Barbara Picht, and Falko Schmieder (eds.). *Das 20. Jahrhundert in Grundbegriffen*. Berlin: Schwabe Verlag. URL: 10.31267/Grundbegriffe (01.10.2025).

[CR21] Graf, Rüdiger 2024b. *Vorhersagen und Kontrollieren. Verhaltenswissen und Verhaltenspolitik in der Zeitgeschichte*. Göttingen: Wallstein Verlag.

[CR22] Gross, Stephen G. 2017. Reimagining Energy and Growth: Decoupling and the Rise of a New Energy Paradigm in West Germany, 1973–1986. *Central European History* (50/4): 514–46.

[CR23] Gross, Stephen G. 2023. *Energy and Power. Germany in the Age of Oil, Atoms, and Climate Change*. Oxford: Oxford University Press.

[CR24] Hacke, Jens 2008. Der Staat in Gefahr. Die Bundesrepublik der 1970er Jahre zwischen Legitimationskrise und Unregierbarkeit. In: Jens Hacke and Dominik Geppert (eds.). *Streit um den Staat. Intellektuelle Debatten in der Bundesrepublik 1960–1980*. Göttingen: Vandenhoeck & Ruprecht: 188–206.

[CR25] Hasenöhrl, Ute and Patrick Kupper 2021. Historicizing renewables: issues and challenges. *History and Technology* (37/4): 397–410.

[CR26] Hesse, Nicole 2016. Windwerkerei. Praktiken der Windenergienutzung in der frühen deutschen Umweltbewegung. *TG Technikgeschichte* (83/2): 125–50.

[CR28] Joerges, Bernward 1988. Large technical systems: Concepts and issues. In: Renate Mayntz and Thomas P. Hughes (eds.), *The Development of Large Technical Systems*, Frankfurt am Main, Boulder, Colorado: Campus Verlag/Westview Press: 9–36.

[CR27] Joerges, Bernward and Marvin E. Olsen 1979. *Policies for Promoting Consumer Energy Conservation: An American-European Perspective. Report transmitted by the Scientific Committee on Problems of the Environment (SCOPE) of the International Council of Scientific Unions (ICSU).* Stockholm: United Nations.

[CR29] Jung, Matthias 1994. *Öffentlichkeit und Sprachwandel. Zur Geschichte des Diskurses über die Atomenergie*. Wiesbaden: VS Verlag für Sozialwissenschaften.

[CR30] Jungk, Robert 1977. *Der Atom-Staat. Vom Fortschritt in die Unmenschlichkeit*. 3rd ed. Munich: Kindler.

[CR31] Kitschelt, Herbert 1984. *Der ökologische Diskurs. Eine Analyse von Gesellschaftskonzeptionen in der Energiedebatte*. Frankfurt am Main: Campus-Verlag.

[CR32] Krause, Florentin, Hartmut Bossel, and Karl Friedrich Müller-Reißmann 1980. *Energie-Wende. Wachstum und Wohlstand ohne Erdöl und Uran. Ein Alternativ-Bericht des Öko-Instituts Freiburg*. Frankfurt am Main: S. Fischer.

[CR33] Leach, Gerald 1979. *A low energy strategy for the United Kingdom*. 2nd print. London: The International Institute for Environment and Development.

[CR34] Lieb, Felix 2022. *Arbeit und Umwelt? Die Umwelt- und Energiepolitik der SPD zwischen Ökologie und Ökonomie 1969–1998*. Munich, Vienna: De Gruyter Oldenbourg.

[CR35] Lönnroth, Måns, Thomas B. Johansson, and Peter Steen 1980. *Solar versus Nuclear. Choosing Energy Futures. A Report*. Oxford, Frankfurt am Main: Pergamon Press.

[CR36] Lovins, Amory B. 1976. Energy Strategy: The Road Not Taken? *Foreign Affairs* (55/1): 65–96.

[CR37] Lovins, Amory B. 1977. *Soft Energy Paths. Toward A Durable Peace*. Harmondsworth, New York: Penguin.

[CR38] Mayntz, Renate and Thomas P. Hughes (eds.) 1988. *The Development of Large Technical Systems*, Frankfurt am Main, Boulder, Colorado: Campus Verlag/Westview Press.

[CR39] Meixner, Horst 1981. Sanfte versus harte Technologien und die Sozialverträglichkeit von Energiesystemen. *Zeitschrift für Umweltpolitik* (3): 331–55.

[CR40] Mettler-Meibom, Barbara and Bertold Wichmann 1982. Informationsstand und Einstellung als Verhaltensregulative. In: VDI, VDE, FfE, IfE, and Socialdata (eds.). *Einfluß des Verbraucherverhaltens auf den Energiebedarf privater Haushalte. Vorträge der Tagung in München am 16. Oktober 1981*. Berlin, Heidelberg: Springer: 73–88.

[CR41] Metzler, Gabriele 2005. *Konzeptionen politischen Handelns von Adenauer bis Brandt. Politische Planung in der pluralistischen Gesellschaft*. Paderborn: F. Schöningh.

[CR42] Meyer, Jan-Henrik and Britta Oertel 2024. *Bürgerdialog Kernenergie (1974–1983) – Staatliches Handeln in der Auseinandersetzung um die nukleare Entsorgung und seine Bedeutung für das heutige Standortauswahlverfahren*. Berlin: Bundesamt für die Sicherheit der nuklearen Entsorgung (BASE).

[CR67] Meyer-Abich, Klaus Michael 1973. Die ökologische Grenze des herkömmlichen Wirtschaftswachstums. In: Henrich von Nussbaum (ed.). *Die Zukunft des Wachstums. Kritische Antworten zum “Bericht des Club of Rome”.* Düsseldorf: Bertelsmann-Universitätsverlag: 163–86.

[CR44] Meyer-Abich, Klaus Michael 1976. Gesamtgesellschaftliche Rahmenbedingungen der Energieversorgung. Kriterien zur Technologiefolgenabschätzung von Energieversorgungssystemen. In: Gesellschaft für Zukunftsfragen (ed.). *Zukunft Energie. Trends und Alternativen. Energiewissenschaftliches Seminar im Rahmen der Deutschen Industrieausstellung*. Berlin: Gesellschaft für Zukunftsfragen: 21–47.

[CR45] Meyer-Abich, Klaus Michael (ed.) 1979a. *Energieeinsparung als neue Energiequelle. Wirtschaftspolitische Möglichkeiten und alternative Technologien*. Munich: Hanser.

[CR46] Meyer-Abich, Klaus Michael 1979b. Soziale Verträglichkeit – ein Kriterium zur Beurteilung alternativer Energieversorgungssysteme. *Evangelische Theologie* (39/1–6): 38–51.

[CR47] Meyer-Abich, Klaus Michael and Bertram Schefold 1986. *Die Grenzen der Atomwirtschaft. Die Zukunft von Energie, Wirtschaft und Gesellschaft*. Munich: Beck.

[CR48] Meyer-Renschhausen, Martin 1981. *Das Energieprogramm der Bundesregierung. Ursachen und Probleme staatlicher Planung im Energiesektor der BRD*. Frankfurt am Main, New York: Campus-Verlag.

[CR49] Möckel, Benjamin 2022. Auf der Suche nach ökonomischen Alternativen. Ernst Friedrich Schumachers »Small is Beautiful« (1973). *Zeithistorische Forschungen* (19/3): 600–07.

[CR50] Möckel, Benjamin 2024. Die Erfindung des moralischen Konsumenten. Globale Produkte und politischer Protest seit den 1950er Jahren. Göttingen: Wallstein Verlag.

[CR51] Oberloskamp, Eva 2025. *Energie, Ökologie, Markt. Bundesdeutsche und britische Neuansätze in den 1970er und 1980er Jahren*. Frankfurt am Main, New York: Campus-Verlag.

[CR52] Oesterwind, Dieter, Ortwin Renn, and Alfred Voß 1980. *Sanfte Energieversorgung. Möglichkeiten – Probleme – Grenzen*. Jülich: Kernforschungsanlage Jülich.

[CR53] Renn, Ortwin, Gabriele Albrecht, Ulrich Kotte, Hans Peter Peters, and Hans U. Stegelmann 1985. *Sozialverträgliche Energiepolitik. Ein Gutachten für die Bundesregierung*. Munich: High-Tech Verlag.

[CR54] Ruske, Barbara and Dieter Teufel 1980. *Das sanfte Energie-Handbuch. Wege aus der Unvernunft der Energieplanung in der Bundesrepublik*. Reinbek bei Hamburg: Rowohlt.

[CR55] Saretzki, Thomas 2005. Politikberatung durch Bürgergutachten? Konzept und Praxis des „kooperativen Diskurses“. *Zeitschrift für Parlamentsfragen* (3): 630–48.

[CR56] Schaefer, Helmut 1980. Wege und Techniken zur rationelleren Energiebedarfsdeckung. In: Helmut Schaefer (ed.). *Struktur und Analyse des Energieverbrauchs der Bundesrepublik Deutschland*. Gräfelfing/Munich: Technischer Verlag Resch KG: 183–207.

[CR57] Schmitt, Dieter, Heinz Jürgen Schürmann, and Wolf Pluge 1976. *Zur Notwendigkeit einer staatlichen Energieeinsparungspolitik. Ein kritischer Ansatz*. Munich: Oldenbourg.

[CR58] Schüring, Michael 2015. *»Bekennen gegen den Atomstaat«. Die evangelischen Kirchen in der Bundesrepublik Deutschland und die Konflikte um die Atomenergie 1970–1990*. Göttingen: Wallstein Verlag.

[CR59] Seefried, Elke 2015. *Zukünfte. Aufstieg und Krise der Zukunftsforschung 1945–1980*. Berlin, Boston: De Gruyter Oldenbourg.

[CR60] Trischler, Helmuth and Robert Bud 2018. Public Technology: Nuclear Energy in Europe. *History and Technology* (34/3–4): 187–212.

[CR61] Turnbull, Thomas 2017. *From Paradox to Policy: The Problem of Energy Resource Conservation in Britain and America, 1865–1981*. PhD Thesis, University of Oxford.

[CR62] Uekötter, Frank 2022. *Atomare Demokratie. Eine Geschichte der Kernenergie in Deutschland*. Stuttgart: Franz Steiner Verlag.

[CR63] Vowe, Gerhard 1991. *Technik im parlamentarischen Diskurs. Die Enquete-Kommissionen des Deutschen Bundestages zum Verhältnis von Technik und Politik*. Habilitation Thesis, Technische Hochschule Darmstadt.

[CR64] Wahl, Bruno 1977. *Technologien zur Einsparung von Energie. Studie ET-5012 A im Auftrag des Bundesministers für Forschung und Technologie*. Kurzfassung und Erläuterung des technischen Teils der Studie. Munich: Zentralstelle für Luft- und Raumfahrtdokumentation und -information der Deutschen Forschungs- und Versuchsanstalt für Luft- und Raumfahrt.

[CR65] Wiesenthal, Helmut 1982. Alternative Technologie und gesellschaftliche Alternativen. Zum Problem der Technikwahl. *Technik und Gesellschaft. Jahrbuch* (1): 48–78.

[CR66] Zachmann, Karin 2012. Past and Present Energy Societies. How Energy Connects Politics, Technologies and Cultures. In: Nina Möllers and Karin Zachmann (eds.). *Past and Present Energy Societies. How Energy Connects Politics, Technologies and Cultures*. Berlin: De Gruyter: 7–41.

